# Human Bocavirus in Infants, New Zealand

**DOI:** 10.3201/eid1311.070793

**Published:** 2007-11

**Authors:** Natalie Redshaw, Catherine Wood, Fenella Rich, Keith Grimwood, Joanna R. Kirman

**Affiliations:** * Malaghan Institute of Medical Research, Wellington, New Zealand; † University of Otago, Wellington, New Zealand

**Keywords:** Human bocavirus, bronchiolitis, New Zealand, parvoviridae, respiratory illness, diarrhea, letter

**To the Editor**: In 2005, a parvovirus, subsequently named human bocavirus (HBoV), was discovered in respiratory samples taken from infants and children hospitalized at Karolinksa University Hospital, Sweden, with lower respiratory tract infection (*1*). HBoV has since been identified in infants and children with respiratory illness in >17 countries, at frequencies ranging from 1.5% to >18.0%.

In the past decade New Zealand has experienced increasing bronchiolitis hospitalization rates, currently >70 admissions per 1,000 infants. To determine the contribution of HBoV to New Zealand’s bronchiolitis disease prevalence, we tested samples collected from infants hospitalized with community-acquired bronchiolitis (*2*) during 3 consecutive winter epidemics (June to October, 2003; July to October, 2004; and June to October, 2005) in Wellington, NZ, for HBoV by PCR. The Central Regional Ethics Committee approved the study. Written, informed consent was obtained from the parent or guardian.

Demographic, clinical, and laboratory data were collected during hospitalization. Ethnicity of those who ascribe to >1 group was determined by using a national census method that prioritizes ethnicity as follows: Māori>Pacific>Other>New Zealand European. Oxygen requirement was determined to be the best measure of bronchiolitis severity (*2*). Infants who needed assisted ventilation or continuous positive airway pressure were classified severe; those who required oxygen supplementation, moderate; and infants who were hospitalized but did not require supplemental oxygen, mild.

Nucleic acid was extracted from thawed nasopharyngeal aspirates (stored at 80°C) by using a High Pure Viral Nucleic Acid kit (Roche Diagnostics, Auckland, NZ). The HBoV nonstructural protein (NP*-*1) gene was amplified by using primers 188F (5′-GAGCTCTGTAAGTACTATTAC-3′) and 542R (5′-CTCTGTGTTGACTGAATACAG-3′) (*1*) with Expand High Fidelity DNA Polymerase (Roche Diagnostics, Basel, Switzerland) for 35 cycles. Products (354 bp) were purified and sequenced from primers 188F and 542R on an ABI3730 Genetic Analyzer by using a BigDye Terminator version 3.1 Ready Reaction Cycle Sequencing kit (Applied Biosystems, Foster City, CA, USA). Sequences were submitted to GenBank under accession nos. EF686006–13.

Alignments of NP-1 gene sequences from nucleotides (nt) 2410–2602, and NP*-*1 predicted amino acid sequences from amino acids (aa) 1–97 were constructed by using ClustalW version 1.83 (available from www.ebi.ac.uk/tools/clustalw/index.html) and compared with HBoV prototype sequences from GenBank (DQ00495-6). Nasopharyngeal aspirates were also screened for respiratory syncytial virus (RSV) by reverse transcription–PCR (RT-PCR) and nested PCR (*3*) and for human metapneumovirus (*4*), influenza A (H1, H3), and influenza B by RT-PCR (*5*).

Eight (3.5%) of 230 samples, collected from infants hospitalized with bronchiolitis during the 2003–2005 winter epidemic seasons, were positive for HBoV. In 5 HBoV-positive infants no other pathogens were identified, but RSV was detected in 3 (Table). The 8 HBoV-positive infants had a median age of 9.5 months, and the male:female ratio was 1:1. The median length of hospital stay was 5.5 (range 1–16) days.

**Table:** Summary of 8 infants with human bocavirus infection hospitalized with bronchiolitis, New Zealand, 2003–2005*

Infant no.	Date admitted	Sex/ age, mo	Ethnicity	Attended daycare?	Length of hospital stay, d	Illness severity	Apnea	Underlying conditions/ comorbitities	RSV subtype	Highest temp., °C	Enteritic symptoms
1	Jul 2003	M/9	Pacific	No	16	Mod	–	–	A	40.1	Diarrhea
2	Aug 2003	F/4	Pacific	No	6	Sev	–	–	B	38.4	Diarrhea
3	Sep 2003	F/11	NZ European	No	1	Mod	–	–	–	38.1	–
4	Sep 2003	F/10	Pacific	No	4	Sev	–	33 weeks’ gestation	–	38.3	Diarrhea
5	Aug 2004	M/8	Pacific	No	2	Mod	–	*Haemophilus influenzae* conjunctivitis	–	37.7	–
6	Jul 2005	M/10	Chinese	No	10	Mod	–	34 weeks’ gestation, repaired esophageal atresia and tracheomalacia	–	37.7	–
7	Aug 2005	F/9	Pacific	No	9	Sev	+	30 weeks’ gestation	A	39.2	–
8	Sep 2005	M/13	NZ European	Yes	5	Mod	–	Hydronephrosis, *Pseudomonas aeruginosa* urinary tract infection	–	37.4	–

*Temp., temperature; Mod, moderate; Sev, severe; –, absent; NZ, New Zealand;+, present.

As expected, because HBoV NP-1 is highly conserved, sequence variation among New Zealand isolates and the prototype Stockholm ST-1 and ST-2 (*1*) NP*-*1 sequences was limited. Alignments of the partial NP*-*1 sequence (nt 2410–2602) of New Zealand isolates with those of ST-1 and ST-2 were identical, except for a G→A change at nt 176 in 2 New Zealand isolates (from infants 5 and 8 years of age), which resulted in a predicted amino acid exchange of S→N at aa 59. In addition, an A→T change at nt 274 in all 8 NZ isolates resulted in a predicted amino acid substitution of T→S at aa 92, a change that has been reported previously in Japanese isolates (*6*).

This study reaffirms previous reports of finding HBoV in a subset of infants with bronchiolitis (*7*). It is also, to our knowledge, the first study of its kind in New Zealand infants, confirming wide distribution of HBoV. In the northern hemisphere, HBoV circulates primarily during the winter months, although it continues circulating until early summer, later than most other seasonal respiratory viruses (*8*). Therefore, this study may underestimate the percentage of New Zealand infants with bronchiolitis whose HBoV test results were positive because sample collection ceased in October (southern hemisphere spring) at the end of the bronchiolitis epidemic. The small number of HBoV-positive infants prevents conclusions concerning ethnicity, coinfection, and bronchiolitis severity.

Although detection of viral nucleic acid by PCR in infants with bronchiolitis does not prove that the virus is the cause of the disease, it raises a hypothesis worthy of investigation. Further studies are required to determine the role of HBoV as a human pathogen. Although coinfection is common, HBoV detection appears to be infrequent in asymptomatic controls (*9*). In our study RSV was detected in 3 (37.5%) HBoV-positive samples. We may have underestimated additional coinfection because we did not test for several respiratory agents, including parainfluenza viruses, rhinoviruses, or the newly discovered coronaviruses.

Finally, HBoV has recently been detected in fecal samples (*10*). Because 3 HBoV-positive infants had diarrhea in addition to bronchiolitis, knowing prevalence of HBoV in fecal specimens from asymptomatic New Zealand children and in those with acute gastroenteritis would be of interest.
